# Comprehensive dataset of features describing eye-gaze dynamics across multiple tasks

**DOI:** 10.1038/s41597-026-06754-x

**Published:** 2026-02-07

**Authors:** Rujeena Mathema, Shamimeh M. Nav, Shailendra Bhandari, Manoj Regmi, Pedro G. Lind, Anis Yazidi, Pedro Lencastre

**Affiliations:** 1 Department of Computer Science, OsloMet - Oslo Metropolitan University, P.O. Box 4 St. Olavs plass, 0130 Oslo, Norway; 2 OsloMet Artificial Intelligence Lab, Stensberggata 29, N-0166 Oslo, Norway; 3 School of Economics, Innovation and Technology, Kristiania University of Applied Sciences, 0153 Oslo, Norway; 4 Simula Research Laboratory, Numerical Analysis and Scientific Computing, Oslo, 0164 Norway; 5 Department of Informatics, University of Oslo, 0316 Oslo, Norway

**Keywords:** Predictive markers, Psychophysics

## Abstract

We present a comprehensive dataset collected from 251 participants, which includes diverse features characterizing eye-gaze dynamics. Participants performed several tasks per session, typical in eye-tracking research: vanishing saccade, cued saccade, flickering cross, rotating ball, and free viewing. Covering these experimental paradigms, the dataset enables analysis of human eye phenomena such as oculomotor control and perceptual processing. As features, the dataset includes timestamped gaze coordinates, pupil sizes, and event classifications, labelled as fixations, saccades, or blinks. All the data were recorded using the EyeLink Portable Duo eye-tracker hardware at 1000 Hz, and processed from raw EyeLink Data Format (EDF) files into structured files via an automated pipeline. The dataset was anonymized according to the Norwegian Agency for Shared Services in Education and Research (SIKT) ethical standards. Possible applications of these datasets are the study of visual attention, cognitive science, and assistive technology.

## Background & Summary

Eye-gaze dynamics are complex, reflecting the intricate interplay of sensory, neural, and cognitive processes that govern visual attention. To explore these dynamics, we have collected data from a series of tasks designed to capture diverse aspects of eye movements, including fixations, saccades, blinks, and pupil responses. This dataset has been pre-processed and structured to facilitate educational and research applications, offering a valuable resource for studying visual attention across various contexts.

Eye tracking provides a powerful method for recording eye movements and gaze locations, enabling researchers to study visual attention over time and across tasks. The field traces its origins to early studies in the 20^th^ century, with pioneers like Buswell and Yarbus demonstrating that eye movements reflect underlying perceptual and cognitive strategies^1,2^. Since then, eye-tracking technology has evolved significantly, transitioning to modern video-based systems that use infrared light to track gaze in real time with high precision, often exceeding 1000 Hz sampling rates and achieving spatial accuracy below 0.5 degrees^3,4^. These advancements have driven a surge in eye-tracking research across disciplines such as psychology, cognitive science, and computer vision^5,6^.

The human eye captures light to enable detailed perception, with eye movements facilitating interaction with the visual environment. These movements are categorized into types such as fixations, saccades, smooth pursuit, vergence, optokinetic nystagmus, and the vestibulo-ocular reflex and they work together to support vision^3,5^. Fixations, periods of relative stability, allow detailed information extraction via the fovea, accompanied by involuntary micro-movements like microsaccades, drifts, and tremors that prevent visual fading^7,8^. Saccades, rapid movements reaching velocities up to 500 degrees per second, reposition gaze to scan the environment, with visual input suppressed during motion to maintain perceptual stability^3,9^. These movements provide critical insights into cognitive processes, with fixation duration and saccade latency serving as indicators of attention, mental effort, and decision-making^8,10^. Microsaccades are tiny, involuntary eye movements that occur during a period of fixation to prevent visual fading and refresh the image on the retina. They occur several times a second, and are essential for visual processing, attention shifts, and maintaining clear vision^11^. Smooth pursuits, on the other hand, are voluntary eye movements that are used to track a moving target with high precision, ensuring the image remains on the center of the eye (fovea) for clear vision^12^.

Eye-tracking metrics such as gaze coordinates, fixations, saccades, blinks, and pupil size are widely used to study visual attention, emotional arousal, and cognitive workload^13^. For instance, longer fixation durations often indicate increased cognitive processing demands^14^, while pupil size reflects cognitive load and emotional states^9,15^. The tasks in this dataset, including paradigms like the Vanishing Saccade, Cued Saccade, Flickering Cross, Rotating Ball, and Free-Viewing tasks, are designed to elicit specific eye movement patterns, enabling analysis of oculomotor control, visual attention, and perceptual processing^3^. These paradigms, rooted in historical studies like the Posner cueing task^16^ and structure-from-motion illusions^17^, provide a diverse dataset for robust analysis. As far as the authors are aware, this is the first open source dataset that not only investigates aspects of human attention but also cognition at a subliminal level associated with eye-tracking and complemented by extensive free-viewing tasks.

Despite advancements, eye-tracking solutions face challenges, including high costs, invasive hardware, and real-world inaccuracies, limiting accessibility with standard devices like smartphones or webcams^18^. Datasets like GazeCapture address these issues by enabling mobile eye-tracking with nearly 1,500 participants, supporting models like iTracker, which achieves gaze prediction errors of 1.04 cm on phones using only eye and face crops^19^. Existing eye-tracking datasets, such as GazeBaseVR^20^, EyeT4Empathy^21^, and TüEyeQ^22^, vary in sampling rates (e.g., 250 Hz to 120 Hz) and often lack standardized structures, complicating cross-study comparisons. The dataset presented here builds on these foundations, offering a pre-processed, task-focused collection to support research and education in visual attention, and perceptual processing.

## Methods

### Hardware

#### The eyelink duo

**Fig. 1:**
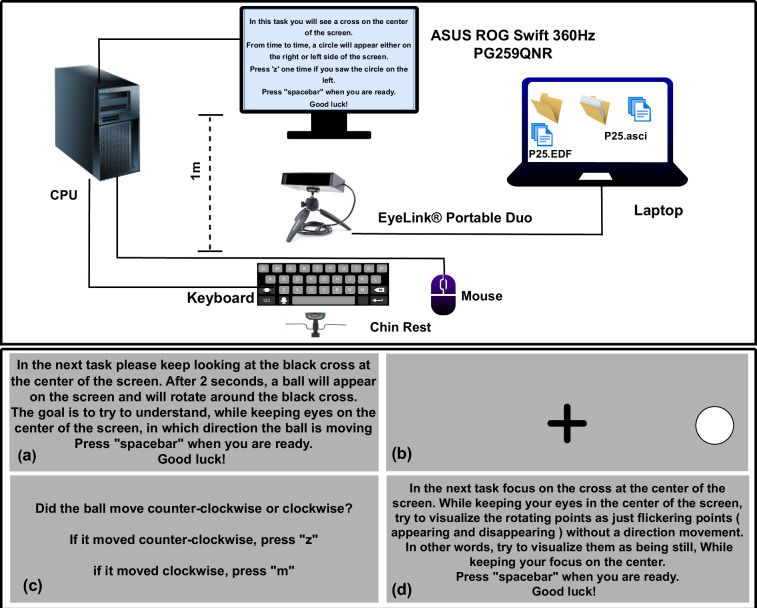
Experimental setup and visual stimuli for the eye-tracking data collection. (Top): Hardware configuration showing the ASUS ROG Swift 360Hz display (1 m viewing distance), EyeLink Portable Duo eye tracker (1000 Hz sampling rate), CPU, laptop for stimulus presentation, keyboard, mouse, and chin rest for head stabilization. (Bottom): The Rotating Ball task, one of several tasks described in Section *Experimental protocol*: (a) task instructions with central fixation cross, (b) rotating ball stimulus, (c) response prompt for rotation direction discrimination, and (d) instructions for the perceptual reversal condition.

The EyeLink duo^23^, shown in Fig. 1, is an advanced eye-tracking device that delivers high-precision measurements of gaze position and pupil size using pupil-corneal reflection tracking, achieving average accuracy down to 0.25°-0.5° and spatial resolution of 0.01°. This system ensures reliable and detailed data capture, even under demanding conditions, making it a versatile tool for various experimental setups. It supports multiple configurations, including remote, head-free, and head-stabilized modes, each equipped with specialized algorithms to minimize noise and enhance accuracy. The EyeLink duo achieves sampling rates of up to 2000 Hz in head-stabilized mode and 1000 Hz in head-free mode, enabling precise tracking of rapid eye movements. Its consistent binocular sampling capability facilitates in-depth analysis of complex eye behaviors, such as pupillometry, microsaccade detection, and gaze-contingent paradigms, with the flexibility to operate in either monocular or binocular tracking modes. Data is efficiently stored in the EDF, a binary format that supports streamlined storage and processing^24^. With its adaptability, compactness, and high accuracy, the EyeLink duo is particularly valuable for researchers investigating eye movement dynamics, visual attention, and related fields.

#### ASUS ROG Swift 360Hz PG259QNR

The ASUS ROG Swift 360Hz PG259QNR, as shown in Fig. 1, is a high-performance gaming monitor designed for professional esports athletes, featuring a 24.5-inch Fast IPS display with a Full HD resolution of 1920 × 1080 pixels for sharp visuals ideal for competitive gaming. Its standout feature is a 360 Hz refresh rate, which ensures ultra-smooth visual motion and significantly reduces motion blur, complemented by the NVIDIA® G-SYNC® processor that synchronizes the display’s refresh rate with the graphics card to eliminate screen tearing and enhance gameplay fluidity. The monitor also integrates the NVIDIA® Reflex Latency Analyzer, enabling precise measurement and optimization of system latency, which allows gamers to fine-tune their setups for minimal latency and maximum responsiveness–a critical advantage in competitive scenarios. Additionally, ASUS Fast IPS technology delivers a rapid 1 ms gray-to-gray response time, ensuring graphics remain clear and free of ghosting effects during high-speed action sequences at elevated frame rates^25^. Engineered to meet the rigorous demands of professional gaming, the ASUS ROG Swift 360Hz PG259QNR combines cutting-edge display technologies with performance-oriented features, providing an optimal balance of speed, visual clarity, and responsiveness. This makes it highly suitable for eye-tracking applications, where high refresh rates and low latency are essential for accurately capturing rapid eye movements and ensuring a seamless user experience. In gaze-contingent and other high-temporal-precision paradigms, a “high refresh rate” is often taken to mean at least 120 Hz^26^.

### Experimental protocol

Figure 1 illustrates the detailed experimental setup designed to investigate whether people can perceive visual information at extremely high frequencies. At the center of this setup are two main devices: the ASUS ROG Swift 360Hz PG259QNR gaming monitor and the Eyelink Portable Duo eye-tracker with sampling rate of 1000 Hz. These devices are connected to a central computer (CPU), along with standard devices such as a keyboard, mouse, and a laptop dedicated to running the eye-tracking software. The CPU is essential for running the experiment’s software, presenting visual stimuli to participants, and ensuring the precise timing of these presentations. Participants use the keyboard and mouse to respond to the visual tasks, allowing accurate recording of reaction times and other performance measures. The laptop specifically manages the Eyelink Portable duo eye-tracker software, performing tasks such as calibrating the eye-tracker, continuously monitoring eye movements, and collecting detailed data throughout the experiment. Proper positioning of equipment is important to maintain accuracy and reliability. It is recommended that the ASUS PG259QNR monitor be placed about one meter away from the participant to provide the best viewing experience and consistent testing conditions. The Eyelink Portable Duo eye tracker should be mounted securely about 45 centimeters from the participant’s eyes using a stable chin rest. This setup helps ensure stability, reduces measurement errors, and improves the accuracy of the eye-tracking data. This experimental arrangement effectively combines advanced monitor technology with precise eye-tracking equipment. It enables researchers to accurately determine the visual perception abilities of participants, particularly in detecting visual stimuli at high refresh rates.

#### Task 1: Vanishing saccade task

The vanishing saccade task was designed to evaluate participants’ ability to detect brief visual stimuli, focusing on how detection performance varies with stimulus duration rather than detailed eye movement metrics such as saccade accuracy or reaction time. The visual stimuli are presented so briefly that it disappears before a onset of a saccade, this forces the brain to rely on the vanishing stimuli trace for detection. This task is based on the classic psychophysical paradigms assessing temporal summation and detection thresholds for briefly flashed peripheral targets during enforced central fixation^27,28^. The three-alternative forced-choice response format (left, right, or none) aligns with signal detection theory approaches to minimise bias in threshold measurement^29^. At the start of each trial, a fixation cross is presented at the center of the screen, and participants are instructed to maintain their gaze on it. Following a random delay drawn from the absolute value of a normal distribution, |*N*(0, 0.5)| seconds, a white circle briefly appears either to the left or right of the cross. Participants indicate the circle’s location by pressing the Z key for the left side, the M key for the right side, or Spacebar if no circle is perceived. The stimulus duration is determined by the formula *N*/240 seconds, where *N* ∈ {4, 2, 1}, corresponding to 4, 2, or 1 frame(s) at a screen refresh rate of 240 Hz. Thus, the durations are 16.67 ms, 8.33 ms, and 4.17 ms, respectively, making detection progressively more challenging as *N* decreases. The rational behind this choice of values is as follows^30^: (i) typical critical flicker fusion thresholds are within the mid-point of this range ([60,240] Hz), (ii) by choosing a geometrical progression of different frequencies we cover that range with just a few points, representing low, medium and high frequencies.

Each participant completes 24 trials, with eight trials at each of the three durations. Responses of participants are recorded as 1, if they perceived the correct position of the circle; -1 if incorrect; and 0 if no circle was perceived.

The primary objective of this task is to quantify how detection accuracy changes with decreasing stimulus duration. The experimental setup included the fixation period, stimulus presentation, and participant response. Detection accuracy is measured as the probability of correctly identifying the circle’s location, denoted by *α*. This probability is expected to decrease with shorter stimulus durations, following the relationship

1 $${\alpha }_{60} > {\alpha }_{120} > {\alpha }_{240},$$

where the subscripts reflect the effective frequency of the stimulus presentation (in Hz) based on the number of frames (e.g., 240 Hz for 1 frame). Higher *α* values indicate better detection performance, with the trend reflecting the increased difficulty of perceiving briefer stimuli.

#### Task 2: Cued saccade task

The cued saccade task examines whether brief, subliminal cues can decrease the time needed to detect and respond to a visual target by comparing reaction times across cued and non-cued conditions. The term cued saccade in this task refers to the congruent nature of the task, which follows the Posner cueing paradigm, where a brief cue is shown before a target that requires either a manual or ocular response. It demonstrates that even without shifting their eyes, individuals react much more quickly to a target once their attention has already been focused on its position^16^. In this task, participants fixate on a central cross throughout each trial, during which a white circle (target) appears on either the left or right side of the screen for 1 second. This task consists of 30 trials in total, 15 for each non-cued and cued trial. The task alternates between non-cued and cued trials to evaluate the effect of cues on response latency.

Non-cued trials begin with the fixation cross, followed by a random delay sampled from |*N*(0, 0.5)| seconds, after which the white target appears. These trials last approximately 1 second. In cued trials, a gray cue precedes the target: after the fixation cross, the cue is presented on one side for *N*/240 seconds (*N* ∈ {4, 2, 1}, corresponding to 16.67 ms, 8.33 ms, or 4.17 ms at a 240 Hz refresh rate) following a delay sampled from |*N*(0, 0.5)| seconds.

After the cue disappears, the fixation cross persists for 0.5 seconds, followed by another delay from the same distribution, before the white target appears on the same side as the cue for 1 second. Cued trials last approximately 2 seconds. Each participant completes 15 cued trials, with 5 trials for each *N* value.

Reaction times are quantified using latency measures defined as

2 $$\Delta {t}_{R1}=t({s}_{1})-t({o}_{1}),\quad \Delta {t}_{R2}=t({s}_{2})-t({o}_{2}),$$

where *t*(*s*_1_) and *t*(*s*_2_) represent the saccade onset times, and *t*(*o*_1_) and *t*(*o*_2_) denote the target onset times in the non-cued and cued conditions, respectively. In cued trials, the cue onset is recorded as *t*(*x*_2_). The expected outcome is

3 $$\Delta {t}_{R1} > \Delta {t}_{R2},$$

suggesting that a preceding cue reduces reaction time. However, as indicated by the results of Task 1 (vanishing saccade task), when the cue duration is 1 frame (4.17 ms), it may be subliminal, potentially leading to reaction times similar to those in non-cued trials.

#### Task 3: Flickering cross-task

The flickering cross task assesses perceptual sensitivity to brief luminance changes without requiring eye movements, aiming to determine the display rate at which flickering becomes indistinguishable from a steady image. Each trial presents two crosses–one on the left and one on the right side of the screen. One cross flickers on and off at intervals of *k* frames (*k* ∈ {8, 4, 2}), while the other remains continuously visible with an opacity of 1/*k* to match the average luminance of the flickering cross. The flicker frequency increases across trials by decreasing *k*. Each trial lasts 1 second, after which participants indicate the flickering cross by pressing Z for the left cross, M for the right cross, or the Spacebar if no flicker is perceived. Responses of participants are recorded as 1, if they perceived the correct position of the flickering cross, -1 if incorrect, and 0 if no flickering cross was perceived. The positions of the flickering and non-flickering crosses are randomized to avoid spatial bias. To ensure that the average brightness of both crosses is exactly the same, the steady cross is set to 1/*k* opacity. This guarantees that participants are selecting based on flicker rather than their brightness. Detection accuracy is analyzed across decreasing *k* values to evaluate performance at higher temporal frequencies. The probability of correctly identifying the flickering cross, denoted *α*_*k*_, is expected to decrease with increasing flicker frequency, following the trend

4 $${\alpha }_{30} > {\alpha }_{60} > {\alpha }_{120},$$

reflecting the reduced ability to detect flicker at faster presentation rates.

#### Task 4: Rotating ball task

The rotating ball task investigates how gaze behavior influences the perception of rotation in an ambiguous motion stimulus, focusing on observers’ ability to identify and intentionally reverse the perceived direction of a rotating ball near a central fixation point. The display consists of a fixation cross at the screen’s center and a ball rotating at a fixed angular velocity *k*. The task comprises two trial types, both requiring central fixation throughout. In the first trial type, participants are instructed to focus at the central fixating cross and report the perceived rotation direction of the ball, pressing z for counterclockwise or m for clockwise motion, and space if they are unsure. In the second trial type, participants attempt to reverse the perceived rotation direction, indicating success with Z or persistence of the initial direction with M. Responses of participants in the first trial are recorded as 1 if they perceived the direction to be counterclockwise; 0, if clockwise; and -1, if they are unsure. In the second trial, the responses are recorded as 1 if they could reverse the perceived direction of rotation, 0 if they could not. Since the stimulus is physically unchanging, the task highlights internally driven perceptual dynamics rather than stimulus-based motion. In order to achieve this volitional reversal, the participants were asked to purposefully suppress global motion signals in favor of local temporal transients by top-down attentional modulation^31^. Poom *et al*.^32^ recently observed that the reversals in perceived depth while observing a wobbling structure form motion are driven by top-down approaches. Performance is assessed based on the frequency and success of perceptual reversals and the consistency of reported rotation directions across trials, isolating gaze-independent perceptual mechanisms.

#### Task 5.1: Free-viewing with random pixel arrays

**Fig. 2:**
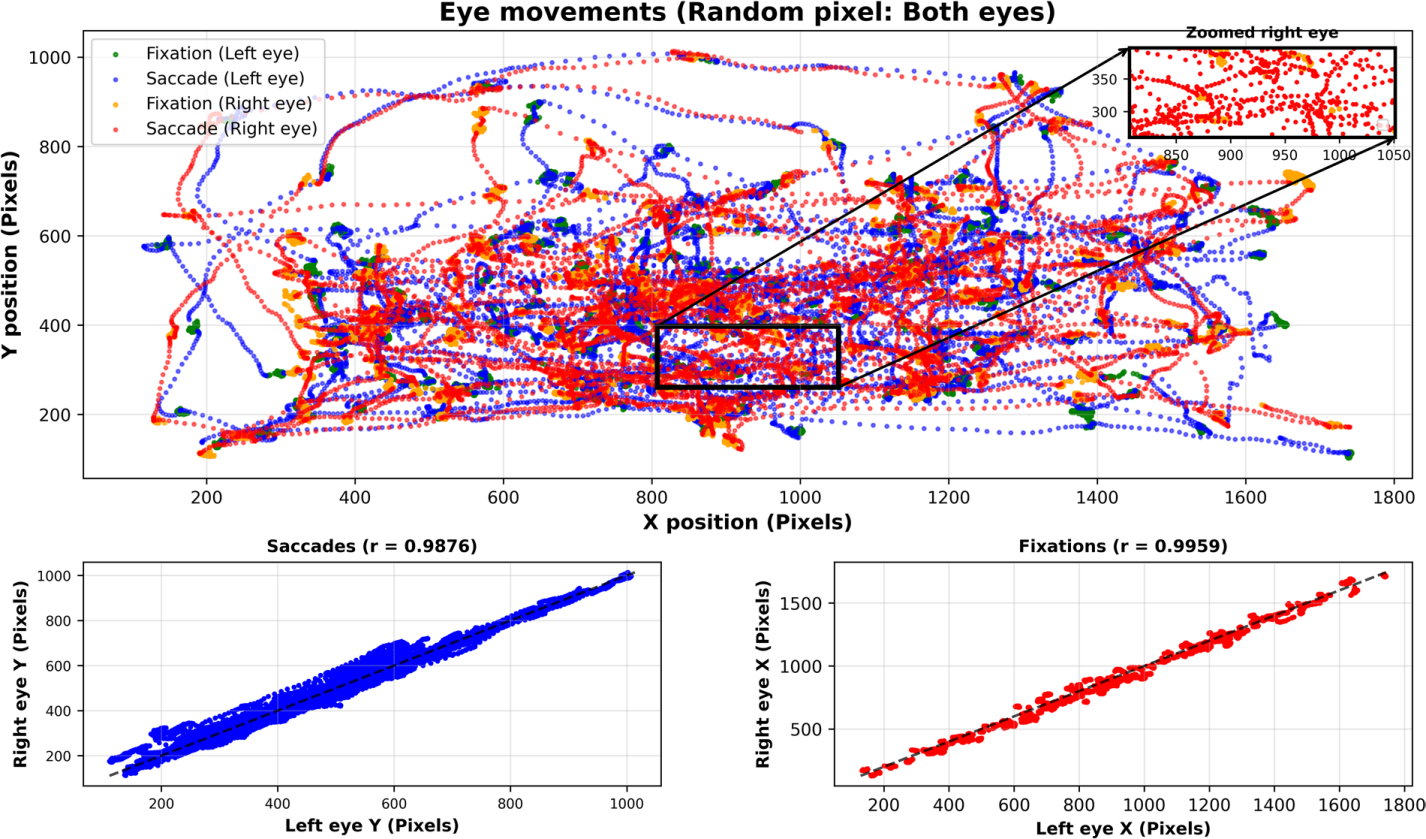
Eye-tracking data from the random pixel task. (Top) The spatial distribution of fixations and saccades for both eyes, with color coding indicating eye and movement type. The black rectangle marks the central region, which is enlarged in the inset to display right-eye fixations (orange) and saccades (red) in greater detail. (Bottom) Scatter plots of left- versus right-eye coordinates, demonstrating strong interocular correspondence for saccades (*r* = 0.9876) and fixations (*r* = 0.9959).

The free-viewing with random pixel arrays task examines eye movement behavior during passive viewing of images lacking coherent structure, capturing natural fixations and saccades without semantic or goal-directed content. Participants view a sequence of 3 images composed of randomly arranged colored pixels on a monitor, with each image displayed for 60 seconds. Participants are instructed to find the hidden patterns in the images. Eye-tracking data, including fixation positions, durations, and saccadic movements, are collected throughout the session. Although the images lack recognizable content, variations in local contrast, brightness, or color may influence gaze direction. Consequently, fixation patterns are expected to vary in density and distribution, reflecting the impact of low-level visual properties on attention allocation Fig. 2 illustrates the random pixel task, with the top panel showing the abstract visualization of eye-tracking data and the lower panels comparing left- and right-eye positions for correlation analysis. Strong correlations were observed between the two eyes, with saccades (bottom left) yielding *r* = 0.9876 and fixations (bottom right) yielding *r* = 0.9959.

These findings provide a framework for investigating visual attention driven exclusively by bottom-up features, assisting in the development of computational models of saliency and early-stage perceptual processing.

#### Task 5.2: Free-viewing with “Where’s Waldo?” images

The free-viewing with “Where’s Waldo?” images task explores natural eye movement behavior during passive observation of visually complex scenes, using detailed “Where’s Waldo?” illustrations to elicit rich gaze patterns with explicit search instructions. Participants view a series of 9 images on a display equipped with an eye-tracking system, with each image presented for 45 seconds. They are instructed to find certain characters and objects in the images, and eye movement data, including fixations, saccade trajectories, and fixation duration, are recorded continuously. The resulting fixation maps and scanpaths offer insights into how visual attention is distributed in cluttered environments and contribute to the development of models of human visual search and scene perception. This “Where’s Waldo” visual search paradigm with crowded scene drawings constitutes a well-established experimental approach to measure how observers efficiently locate target objects amidst visual clutter through integration of bottom-up and top-down attentional signals^33^, providing rich scanpath data for analyzing complex visual search behavior in naturalistic viewing contexts.

### Ethical requirements

Participants were invited to join the experiment via email, which provided an overview of the study’s purpose. They were informed of their right to withdraw at any time without any consequences and were offered a gift card as a token of appreciation upon successful completion. Before participation, all participants signed a consent form. Ethical considerations were rigorously followed in this study. Informed consent was obtained from all participants, with clear communication regarding the study’s aims and procedures. Participants explicitly consented to data sharing for research purposes following full anonymization at the end of the project, which includes deletion of the identification key and ensures that data subjects are not identifiable in publications. Participant confidentiality was prioritized by anonymizing personal data to ensure privacy and prevent identification. Participants were assured of their right to withdraw at any stage without penalty.

Ethical requirements are fulfilled under the approval of the Norwegian Agency for Shared Services in Education and Research (SIKT), reference number 129768, and also under the approval of the Regional Committees for Medical and Health Research Ethics in Norway (REK), reference number 617762. Additionally, the shared forms with all participants were done through the platform: The main website of this platform can be accessed at https://nettskjema.no/user, which assures data protection and anonymity following Norwegian ethical requirements. The participants were requested to sign a consent form stating the purpose of the experiment and the anonymous nature of the data. They were also informed about the voluntary nature of the experiment.

### Experimental data collection and processing

This section describes the methodology for collecting and processing experimental eye-tracking data, which underpins the analyses presented in this section. Eye-tracking data were recorded using the EyeLink system during a series of controlled experimental tasks designed to capture diverse gaze features, including fixations, saccades, blinks, and pupil responses. No missing data, other than blinks, are registered. The raw data, initially stored in the EDF, were processed using the automated pre-processing pipeline, transforming proprietary EDF files into structured, task-specific CSV datasets enriched with trial-level metadata and cleaned of common artefacts.

Eye-tracking data collection involved participants performing tasks under controlled conditions, with each task-trial combination capturing synchronized gaze data for both eyes using the EyeLink system’s high-precision capabilities. The raw EDF files contained timestamped samples of gaze coordinates, pupil sizes, and eye movement events (saccade and fixation onset), providing a comprehensive record of visual attention dynamics across trials. An excerpt of a converted EyeLink ASCII file, illustrating calibration data and raw gaze samples, is as follows:

**CONVERTED FROM C:\...\ P104_5_1_2024_06_20_10_49.EDF using edfapi

** DATE: Thu Jun 20 04:52:59 2024

** TYPE: EDF_FILE BINARY EVENT SAMPLE TAGGED

** VERSION: EYELINK II 1

** SOURCE: EYELINK CL

** EYELINK II CL v6.14 Mar 6 2020 (EyeLink Portable Duo)

...

MSG 120698 !CAL Calibration points:

MSG 120698 !CAL -36.4, -48.9 -0, 339

MSG 120698 !CAL -55.5, -48.4 -3693, 339

MSG 120698 !CAL -18.3, -48.7 3693, 339

MSG 120698 !CAL -55.8, -57.1 -3814, -2303

MS

MSG 120698 !CAL 0.0, 0.0 0, 0

MSG 120698 !CAL eye check box: (L,R,T,B)

-61 6 -63 6

MSG 120698 !CAL href cal range: (L,R,T,B)

-5721 5721 -3583 4098

MSG 120699 !CAL Cal coeff:(X=a+bx+cy+dxx+eyy,Y=f+gx+goaly+ixx+jyy)

MSG 120700 !CAL CALIBRATION HV9 LR LEFT GOOD

MSG 120700 !CAL CALIBRATION HV9 LR RIGHT GOOD

MSG 137179 VALIDATE LR POINT 0 LEFT at 960,540 OFFSET 0.59 deg. -4.9,27.3 pix.

MSG 137179 VALIDATE LR 4POINT 0 RIGHT at 960,540 OFFSET 0.38 deg. -22.4,4.8 pix..

START 142976 LEFT RIGHT SAMPLES EVENTS

142983 888.3 679.2 599.0 891.9 666.5 534.0 0.0 .....

142984 888.4 678.7 598.0 891.9 666.5 533.0 0.0 .....

142985 888.4 678.1 599.0 891.8 666.6 532.0 0.0 .....

The preprocessing pipeline converts the raw EDF files into structured CSV files, organized by participant-task combinations within task-labeled directories. Each output file adheres to a standardized naming convention for each task encoding metadata, such as Task1_Cued_1_240_0.76_1.66_(0.8,0)_P000_Trial_1.csv, where Task1_Cued indicates the task type (e.g., cued saccade), 4 denotes the step interval in frames, 240 is the frequency of the monitor, 0.7667 and 1.6625 specifies the delays for cue and stimulus in seconds, (0.8,0) is the position of the cue and stimulus, P000 represents the participant ID, and Trial_1 identifies the trial number. The pipeline employs a task extraction process, which segments raw data based on task-specific markers, and a raw-data conversion process, which transforms text-based segments into numerical DataFrames. The resulting CSV files contain synchronized gaze data and event classifications for both eyes, with columns including time for timestamps in milliseconds, L_X, L_Y, and L_Pupil for the left eye’s spatial coordinates and pupil size, and equivalent columns for the right eye. Eye movement states are recorded in L_State and R_State, using integer encodings: 0 for blinks, 1 for fixations, and 2 for saccades, with annotations generated independently per eye. A sample of the processed data is presented in Table 1, illustrating the structured format that facilitates detailed analysis of gaze event distributions, temporal patterns, and bilateral consistency.

**Table 1:** Sample of processed eye-tracking data, showing synchronized gaze coordinates, pupil sizes, and event classifications for both eyes.

Time	L_X	L_Y	L_Pupil	L_X	L_Y	R_Pupil	L_State	R_State
688796169.0	906.0	690.4	556.0	881.9	654.1	589.0	1.0	1.0
688797170.0	905.1	690.4	556.0	882.0	654.6	589.0	1.0	1.0
688865239.0	0.0	0.0	0.0	0.0	0.0	0.0	0.0	0.0
688878523.0	902.9	691.2	555.0	882.2	654.8	589.0	2.0	1.0
688978526.0	902.0	691.3	554.0	882.3	654.4	589.0	2.0	2.0
688996325.0	901.2	691.1	553.0	882.9	654.0	588.0	2.0	2.0
689296175.0	900.8	690.8	553.0	883.5	654.1	588.0	1.0	1.0
691245698.0	901.7	690.8	553.0	884.6	653.1	588.0	2.0	2.0
691547528.0	902.7	690.6	554.0	885.3	652.0	588.0	2.0	1.0
695736512.0	0.0	0.0	0.0	0.0	0.0	0.0	0.0	0.0
696771258.0	904.8	691.1	555.0	885.8	651.5	589.0	1.0	1.0

Legend as follows (see caption of Tab.3): “L_State”, left eye state; “R_State”, right eye state. The states are labelled as 0 (blink), 1 (fixation) and 2 (saccade).

This structured output enables robust statistical analyses and visualizations, as explored in subsequent sections, by providing a clean, standardized dataset that captures the full spectrum of gaze behaviors across experimental conditions.

In the EyeLink system, a message line consists of a timestamp followed by text that marks significant experimental events, such as display changes, participant responses, or system status updates, facilitating synchronization of gaze data with task conditions. These messages are sent by the experimental application (PsychoPy) to the EyeLink tracker to provide metadata for analysis or to denote critical moments in the experiment. The message text occupies the entire line after the timestamp, with any remaining space left blank. Common message types include system-generated events (e.g., calibration/validation results, start/end of trials) and user-defined messages tied to specific task events.

Table 2 summarizes the message types associated with each experimental task in this study, based on the standardized naming convention observed in the processed data (e.g., Task1_Cued). Tasks include vanishing saccade, cued saccade, flickering cross, rotating ball, free viewing with random pixel arrays, and free viewing with “Where’s Waldo?” images, designed to elicit distinct gaze behaviors such as fixations, saccades, and smooth eye movements. Message types are categorized into event markers (e.g., SFIX, EFIX for start and end of fixations; SSACC, ESACC for start and end of saccades; SBLINK, EBLINK for start and end of blinks) and task-specific messages (e.g., trial onset/offset, stimulus presentation). These messages are extracted during the pre-processing pipeline to segment and annotate gaze data accurately.

**Table 2:** Message types associated with experimental tasks, including system-generated eye movement events and task-specific markers.

Task	Message Type	Description
Vanishing Saccade	MSG TRIAL_START	Marks the onset of a trial, including the frequency rate of stimulus.
	START_FIXATION	Start of a fixation event for the specified eye at timestamp stime.
	END_FIXATION	End of a fixation, including duration, average gaze coordinates, and pupil size.
	START_SACCADE	Start of a saccade event.
	END_SACCADE	End of a saccade, including start/end coordinates, amplitude, and peak velocity.
	MSG RESP_ACCURACY	Indicates the response accuracy of identifying the circle position.
	MSG STIM_ONSET	Indicates the condition for presentation of the visual stimulus (e.g., target delay and direction).
	MSG TRIAL_END	Marks the end of a trial, often with response data.
Cued Saccade	MSG TRIAL_START	Marks the onset of a trial, including trial number and condition (e.g., cue direction).
	START_FIXATION	Start of a fixation event for the specified eye at timestamp stime.
	END_FIXATION	End of a fixation, including duration, average gaze coordinates, and pupil size.
	START_SACCADE	Start of a saccade event.
	END_SACCADE	End of a saccade, including start/end coordinates, amplitude, and peak velocity.
	MSG TRIAL_END	Marks the end of a trial, often with response data.
Flickering Cross	MSG TRIAL_START	Marks the onset of a trial.
	START_FIXATION	Start of a fixation event for the specified eye at timestamp stime.
	END_FIXATION	End of a fixation, including duration, average gaze coordinates, and pupil size.
	START_SACCADE	Start of a saccade event.
	END_SACCADE	End of a saccade, including start/end coordinates, amplitude, and peak velocity.
	MSG TRIAL_END	Marks the end of a trial, often with response data.
	MSG RESP_ACCURACY	Indicates the response accuracy of identifying the flickering cross, including the frequency used.
Rotating Ball	MSG TRIAL_START	Marks the onset of a trial.
	START_FIXATION	Start of a fixation event for the specified eye at timestamp stime.
	END_FIXATION	End of a fixation, including duration, average gaze coordinates, and pupil size.
	START_SACCADE	Start of a saccade event.
	END_SACCADE	End of a saccade, including start/end coordinates, amplitude, and peak velocity.
	MSG TRIAL_END	Marks the end of a trial, often with response data.
	MSG RESP_ACCURACY	Indicates the response accuracy of identifying the direction of the ball.
Free Viewing (Random Pixel)	MSG IMAGE_ONSET	Marks the display of a natural image or scene.
	START_FIXATION	Fixation and saccade events as above, capturing exploratory gaze patterns.
	END_FIXATION	
	START_SACCADE	
	END_SACCADE	
	START_BLINK	Start of a blink event.
	END_BLINK	End of a blink, including duration.
	MSG IMAGE_OFFSET	Marks the removal of the image.
Free Viewing ("Where’s Waldo?”)	MSG IMAGE_ONSET	Marks the display of a natural image or scene.
	START_FIXATION	Fixation and saccade events as above, capturing exploratory gaze patterns.
	END_FIXATION	
	START_SACCADE	
	END_SACCADE	
	START_BLINK	Start of a blink event.
	END_BLINK	End of a blink, including duration.
	MSG IMAGE_OFFSET	Marks the removal of the image.

**Fig. 3:**
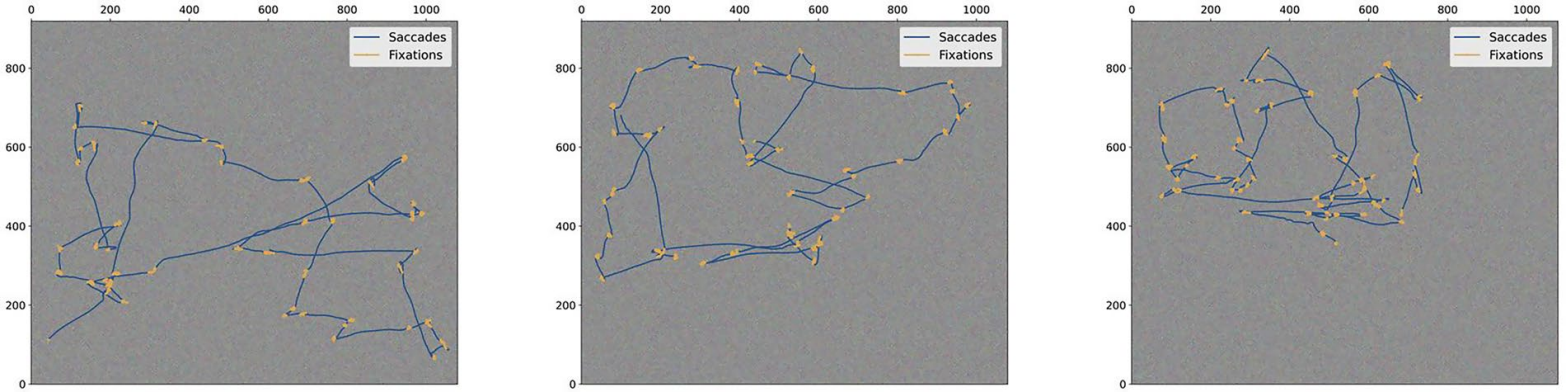
Illustrations of a random participant’s 16s right eye’s gaze trajectory during task 5.1, where saccades are represented in blue color and fixations in yellow.

Saccades and fixations were identified using the SR-Research proprietary algorithm^23^. The algorithm is based on velocity, acceleration, and saccade displacement. We have used the default thresholds: 30°/s for onset and offset gaze velocity, 8000°/s^2^ for onset acceleration, and 0.15° for saccade displacement. Smooth pursuits, microsaccades, and glissades (post-saccadic oscillations)^34^ were not identified by the algorithm. Ocular event identifications were manually inspected in saccade and free-viewing tasks and found to be adequate. Some small dynamical differences between the left and right eyes are expected and are registered in the data. This can cause some discrepancy in the saccade onset / offset time between the left and right eye of, typically, one or two milliseconds. However, for most eye-tracking applications, monocular records are used^35^, so this discrepancy has little practical impact. An illustration of a 16s right eye’s gaze trajectory during task 5.1 can be seen in Fig. 3.

### Data overview: analysis and results

Table 3 summarizes the variables collected in each task, detailing the specific measures and data points gathered throughout the experiment.

**Table 3:** Number of participants, trials, and matrices and variables used for different tasks. Legend as follows: “Part.”, number of participants; “Time”, timestamp; “L_X”, X-position left eye; “L_Y”, Y-position left eye; “L_Pupil”, pupil diameter left eye; “R_X”, X-position right eye; “R_Y”, Y-position right eye; “R_Pupil”, pupil diameter right eye; “N”, frequency (number of frames the stimulus is displayed on the screen); “T_C”, time delay for cue to appear (in seconds); “T_S”, time delay for stimulus to appear (in seconds); “P_C”, position of the cue, (−0.8,0): Left, (0.8,0): Right; “P_S”, position of the stimulus (−0.8,0): Left, (0.8,0): Right; “R_A”, response accuracy.

Task	#Part.	#Trials	Time	L_X	L_Y	L_Pupil	R_X	R_Y	R_Pupil	N	T_C	T_S	P_C	P_S	R_A
1. Vanishing Saccade	149	24^a^	*✓*	*✓*	*✓*	*✓*	*✓*	*✓*	*✓*	*✓*	✗	*✓*	✗	*✓*	*✓*
2. Cued Saccade	184	15+15^b^	*✓*	*✓*	*✓*	*✓*	*✓*	*✓*	*✓*	*✓*	*✓*	*✓*	*✓*	*✓*	✗
3. Flickering Cross	149	9^c^	*✓*	*✓*	*✓*	*✓*	*✓*	*✓*	*✓*	*✓*	✗	✗	✗	✗	*✓*
4. Rotating Ball	184	2^d^	*✓*	*✓*	*✓*	*✓*	*✓*	*✓*	*✓*	✗	✗	✗	✗	✗	*✓*
5.1. Random Pixels	43	3 images	*✓*	*✓*	*✓*	*✓*	*✓*	*✓*	*✓*	✗	✗	✗	✗	✗	✗
5.2."Where’s Waldo?”	139	9 images	*✓*	*✓*	*✓*	*✓*	*✓*	*✓*	*✓*	✗	✗	✗	✗	✗	✗

^a^8 trials for each *N*; *N* ∈ {4, 2, 1}.

^b^5 trials for each value of *N*; *N* ∈ {4, 2, 1}.

^c^3 trials for each value of *k*, where *k* is the number of frames one cross flickers for; *k* ∈ {8, 4, 2}.

^d^Trial 1: Identify direction; Trial 2: Reverse direction.

The automated preprocessing pipeline transforms raw EyeLink ASC files into structured CSV datasets, enabling detailed analysis of gaze dynamics across different experimental tasks. Each participant-task combination yields a CSV file in task-labeled directories, with filenames encoding metadata; TaskA_(taskname)_variables_pID_TrialM.csv; where A indicates the task number, variables indicates the different conditions and variables used in different tasks, pID indicates the participant ID and M indicates the trial number. The data includes include Time (milliseconds), L_X, L_Y, L_Pupil (and right-eye equivalents), and L_State, R_State (0 = blink, 1 = fixation, 2 = saccade), as shown in Table 1. The naming convention for each task with their variables defined in Table 3 is as follows.

- Task1_Non_Cued_(T_C)_(P_C)_pID_TrialM.csv;
- Task1_Cued_(N_240)_(T_C)_(T_S)_(P_S)_(P_C)_pID_TrialM.csv;
- Task2_(N_240)_(R_A)_pID_TrialM.csv;
- Task3_(N_240)_(R_A)_pID_TrialM.csv;
- Task4_(R_A)_pID_TrialM.csv;
- Task5_pID_TrialM.csv;
- Task6_pID_TrialM.csv;

For the Cued Saccade task, certain domination of fixations over saccades can be observed, which is a natural behavior in this task. There exists high binocular coordination between the eyes. In the Free-Viewing with random pixel array task, gaze trajectories exhibit greater time spent on fixation than saccades; this variability is due to unstructured visual stimuli in the task. The gaze trajectories for both eyes maintain strong binocular coordination, as observed in Fig. 2. They show very high correlation in terms of both fixations and saccades. Pupil dynamics during fixations reveal subtle fluctuations, with smoothed pupil size and dilation rates indicating cognitive and attentional changes, while blink distributions show temporal clustering, though high counts (e.g., 51 blinks in a 0.41-second trial) suggest potential detection artifacts. These results demonstrate the pipeline’s ability to produce reliable, analysis-ready data, capturing nuanced gaze behaviors across structured (cued saccade) and unstructured (random pixel) tasks. The dataset supports investigations into oculomotor control and visual attention, complementing the calibration-focused validation in Section *Technical validation* and offering flexibility for analyzing additional tasks (e.g., vanishing saccade, flickering cross). After calibration and validation, no further error correction was implemented.

## Data Records

The dataset is available in the Figshare^36^. The dataset is segregated by participant ID and the version number of the experiment. Each folder consists of an Eyelink Data Format (EDF) and an ASCII (.asc), plain text version of EDF files. The naming convention of the files and folder are *PXXX_Versionnumber_YY_MM_DD_HH_mm*. The version number is simply used to keep track of the routines in the experiments.

## Technical Validation

Technical validation of the eye-tracking data ensures the reliability and accuracy of the recorded gaze measurements, critical for robust analysis of gaze behaviors across experimental tasks. Validation focuses on calibration accuracy, as calibration errors directly impact gaze data quality. Calibration was performed using a standard nine-point grid, with validation conducted immediately after to assess accuracy for both eyes, following best practices in eye-tracking research^37^. The ASCII files contain detailed calibration validation results, exemplified by the following messages:

MSG 137179 !CAL VALIDATION HV9 LR LEFT GOOD ERROR 0.58 avg. 0.99 maxOFFSET 0.28 deg. 5.0,12.4 pix.

MSG 137179 !CAL VALIDATION HV9 LR RIGHT GOOD ERROR 0.44 avg. 0.60 maxOFFSET 0.20 deg. -12.0,1.0 pix.

These messages indicate that validation used a nine-point grid (HV9), with results reported for left (LEFT) and right (RIGHT) eyes. The GOOD ERROR label confirms that the average calibration error (e.g., 0.47^°^ for left eye, 0.57^°^ for right eye) is within the manufacturer’s recommended threshold of 0.5^°^ for high-quality data^24^. The average error represents the mean deviation across validation points, while the maximum error (e.g., 0.81^°^ for left eye, 1.31^°^ for right eye) indicates the largest deviation at any single point. The offset (e.g., 0.29^°^ for the left eye) quantifies additional positional discrepancies in degrees and pixels.

## Usage Notes

### General notes

This eye-tracking dataset was curated to provide researchers with a high-quality, structured resource for studying gaze dynamics across diverse tasks, supporting applications in visual attention, and assistive technology. Recorded using the EyeLink Portable Duo at up to 1000 Hz, the dataset includes gaze coordinates, pupil sizes, and event classifications (fixations, saccades, blinks) for both eyes, processed into CSV files via an automated pipeline. The pipeline corrects common artifacts, such as missing samples or encoding inconsistencies. However, some issues, like high blink counts in certain trials (e.g., 51 blinks in a 0.41-second trial, may reflect detection artifacts due to signal noise or fragmented blink intervals. Raw EDF files and pipeline code are available to allow users to verify or modify preprocessing steps. Researchers should note that event classifications (0 = blink, 1 = fixation, 2 = saccade) are generated independently per eye, with minor mismatches possible due to physiological factors like microsaccades.

### Task-specific considerations

The dataset covers multiple tasks (vanishing saccade, cued saccade, flickering cross, rotating ball, free-viewing with random pixel arrays, and free-viewing with “Where’s Waldo?” images), each eliciting distinct gaze behaviors. For structured tasks like Cued Saccade, fixation-dominated patterns and high bilateral consistency are expected, while Free-Viewing tasks exhibit greater trajectory variability due to unstructured stimuli. Users analyzing pupil dynamics should apply smoothing techniques, as demonstrated in Section *Data overview*, to reduce noise while preserving temporal trends. Task-specific metadata (e.g., trial duration, stimulus onset) is encoded in CSV filenames (e.g., Task1_Cued_1_240_0.76_1.66_(0.8,0)_P000_Trial_1.csv) and message lines (Table 2), aiding synchronization with experimental events.

### Calibration and data gaps

Calibration accuracy is critical for reliable gaze data, with average errors typically below 0.5^°^ (e.g., 0.47^°^ for left eye, meeting EyeLink standards^24^. However, maximum errors occasionally exceed 1^°^ (e.g., 1.31^°^ for the right eye), suggesting potential outliers at specific validation points. Users should inspect calibration validation messages in ASCII files to identify sessions requiring recalibration (see above). Data gaps, primarily due to blinks or hardware interruptions, are minimal but present in some trials, particularly in Free-Viewing tasks with longer durations (e.g., 10 seconds for “Where’s Waldo?” Images). These gaps are not automatically corrected but can be interpolated using standard methods, with raw EDF files provided for reference.

### Analysis recommendations

For advanced analysis of eye-tracking behaviors, the structured CSV format supports tools like pandas and NumPy for processing gaze coordinates, pupil sizes, and event distributions (fixations, saccades, blinks). Classical statistical methods, such as time-series analysis and ANOVA, can quantify saccade latency, fixation duration, and bilateral consistency; however, users should align events for tasks with high variability (e.g., Random Pixel Arrays). Deep learning approaches, including convolutional neural networks (CNNs) for spatial gaze patterns and recurrent neural networks (RNNs) for temporal sequences, enable modeling of complex behaviors like attention shifts. AI methods, such as reinforcement learning for gaze prediction or clustering for fixation pattern detection, can further uncover latent behavioral states. To mitigate blink detection artifacts, applying a minimum duration threshold (e.g.,100 ms) is recommended to filter out noise. Additionally, Ref. ^38–40^ employed classical and quantum GANs to synthesize realistic eye-tracking data for the Waldo task, enhancing model robustness and behavioral analysis.

## Data Availability

The EDF and ASC data files for each participant are available in the Figshare repository^36^ and can be found within the archive eyeGaze_dataSet_OslometEyetracking_Lab.zip.

## Code availability

The Python library used to convert raw EDF recordings into labeled CSV files for the various tasks is provided in the Figshare^36^, under the directory EyeGazeDynamicsPipeline.
